# Who acquires infection from whom and how? Disentangling multi-host and multi-mode transmission dynamics in the ‘elimination’ era

**DOI:** 10.1098/rstb.2016.0091

**Published:** 2017-03-13

**Authors:** Joanne P. Webster, Anna Borlase, James W. Rudge

**Affiliations:** 1 Department of Pathology and Pathogen Biology, Centre for Emerging, Endemic and Exotic Diseases, Royal Veterinary College, University of London, Hatfield AL9 7TA, UK; 2 Communicable Diseases Policy Research Group, London School of Hygiene and Tropical Medicine, Keppel Street, London WC1E 7HT, UK; 3 Faculty of Public Health, Mahidol University, 420/1 Rajavithi Road, Bangkok 10400, Thailand

**Keywords:** pathogen, transmission, multi-host, multi-mode, dynamics, control

## Abstract

Multi-host infectious agents challenge our abilities to understand, predict and manage disease dynamics. Within this, many infectious agents are also able to use, simultaneously or sequentially, multiple modes of transmission. Furthermore, the relative importance of different host species and modes can itself be dynamic, with potential for switches and shifts in host range and/or transmission mode in response to changing selective pressures, such as those imposed by disease control interventions. The epidemiology of such multi-host, multi-mode infectious agents thereby can involve a multi-faceted community of definitive and intermediate/secondary hosts or vectors, often together with infectious stages in the environment, all of which may represent potential targets, as well as specific challenges, particularly where disease elimination is proposed. Here, we explore, focusing on examples from both human and animal pathogen systems, why and how we should aim to disentangle and quantify the relative importance of multi-host multi-mode infectious agent transmission dynamics under contrasting conditions, and ultimately, how this can be used to help achieve efficient and effective disease control.

This article is part of the themed issue ‘Opening the black box: re-examining the ecology and evolution of parasite transmission’.

## 1. Introduction

**Figure 1.**
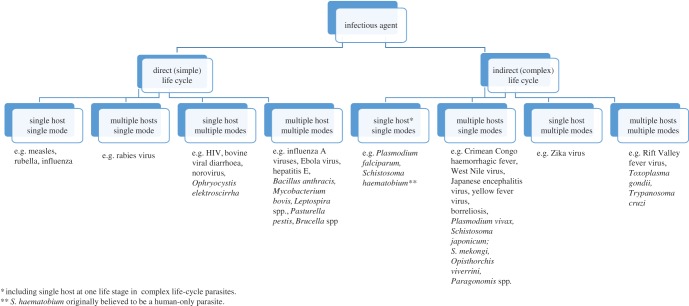
Classification of pathogens by life cycle complexity, number of hosts and number of transmission modes. (Online version in colour.)

**Figure 2.**
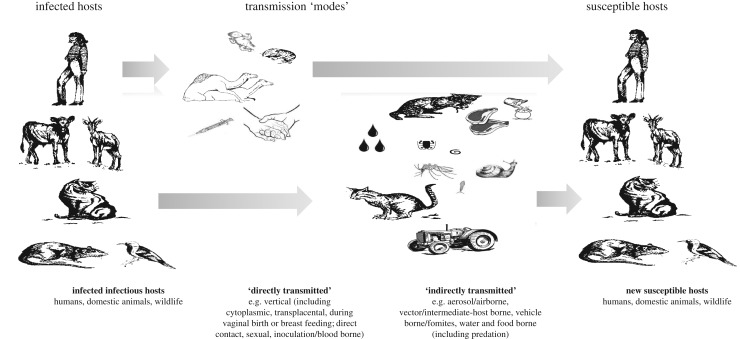
Multiplicity of pathogen transmission pathways and control opportunities. Examples include, infected infectious hosts can be targeted by: test and slaughter of livestock and domestic animals, e.g. FMDV, brucellosis; prophylactic drug treatment to reduce infectious stages transmission to environment, e.g. human MDA for *Schistsosoma* spp., or to offspring, e.g. targeted use of anti-retroviral drugs to reduce the likelihood of vertical transmission of HIV; human use of condoms to prevent sexually transmitted infections, e.g. syphilis, HIV. Indirect environmental and vector-borne transmission can be targeted by: improved health education and sanitation programmes to minimize environmental transmission, e.g. cholera, Guinea worm; improved burial practices to reduce the risk of transmission from people who have died due to, e.g. Ebola; vector and intermediate host control, e.g. malaria, schistosomiasis, dengue. Uninfected hosts can be targeted by: vaccination of uninfected humans to prevent human-to-human direct transmission, e.g. measles, or of livestock or domestic animals to prevent human transmission, e.g. domestic dogs to reduce human cases of rabies due to dog bites, or sheep and cattle to prevent brucellosis transmission to humans; health education.

**Figure 3.**
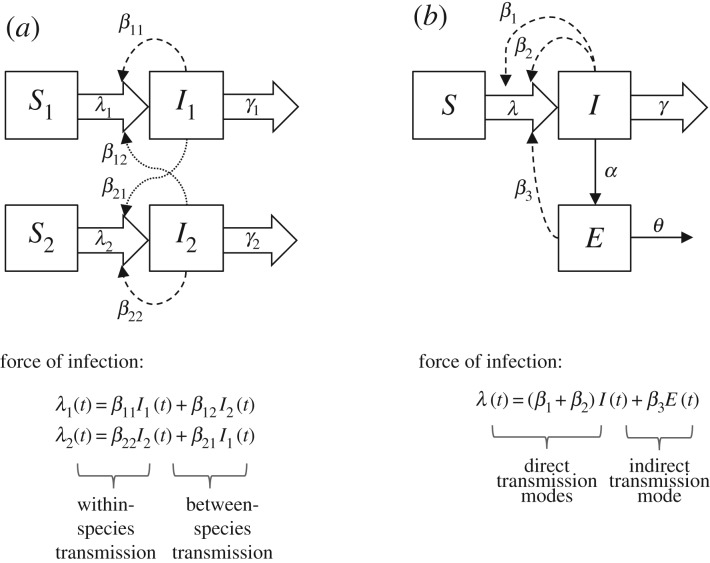
Schematics of simplified models for systems with multiple host species (*a*) and multiple transmission modes (*b*). Model compartments and parameters are defined in table 2. Block arrows represent the flow of individuals between compartments; dashed and dotted arrows represent transmission within and between species, respectively; line arrows show release and decay of indirectly transmitted infective stages. The model in (*a*) depicts a system with two host species, with the force of infection *λ_i_*(*t*) in each host species *i* at time *t* defined as the sum of the forces of infection that can be attributed to transmission from each infected host species *j.* The model in (*b*) shows a single-host system with three modes of transmission, two of which are direct and one of which is indirect via a ‘pool’ of infective stages *E,* which could represent infective stages in the environment, a vector or an intermediate host. In this multi-mode system, the total force of infection is defined as the sum of the forces of infection that can be attributed to each transmission mode, *k*.

Understanding the complex population biology and transmission ecology of multi-host parasites and pathogens has been declared as one of the major challenges of biomedical sciences for the twenty-first century [1], and elucidating and distinguishing between contrasting drivers of disease transmission maintenance and outbreaks is critical in determining policy, targeting interventions and predicting outcomes. Transmission can be defined, at its simplest, as the means by which an infectious agent is passed from an infected host to a susceptible host [2]. Transmission dynamics may involve multiple levels and varying degrees of complexity (figures 1 and 3 and tables 1 and 2), from single-host species in pathogens with direct, or simple, life cycles, such as the human-specific measles virus, to contrasting host stages and species in indirectly transmitted agents with complex life cycles, such as the multiple mammalian definitive hosts (human, domestic and wild animals) and single molluscan intermediate hosts of *Schistosoma japonicum* [9–11]. Within this, many infectious agents are able to use, simultaneously or sequentially, multiple modes of transmission, including but not exclusive to vertical, direct contact, sexual, aerosol, vector-borne and/or food-borne (table 1; figures 1 and 2). The relative importance of different hosts and modes can itself be dynamic, with potential for switches and/or shifts in host range or transmission mode (table 1) of an infectious agent to occur in response to dynamic selective pressures, such as anthropogenic change and disease control interventions [12,13].

**Table 1.** Glossary.

term	definition
definitive host	a host, often but not always a vertebrate, that harbours the pathogen at a mature sexually active phase [2].
elimination	elimination (interruption of transmission) refers to the reduction to zero (or a very low defined target rate) of new cases in a *defined geographical area*. Elimination requires continued measures to prevent re-establishment of disease transmission. Examples: currently targeted in humans—schistosomiasis, lymphatic filariasis, onchocerciasis, leprosy, malaria, *Taenia solium*; currently targeted in animals—brucellosis, bovine tuberculosis.
eradication	the complete and permanent worldwide reduction to zero new cases of an infectious disease through deliberate efforts; no further control measures are required. Examples: achieved in humans—smallpox; achieved in livestock—Rinderpest; currently targeted in humans—Dracunculiasis, poliomyelitis, yaws.
extinction	the specific infectious agent no longer exists in nature or in the laboratory.
host switch	traditionally, host switch is also called host shift as an alternative synonym. However, although often difficult to disentangle without long-term host–parasite data, it is important to distinguish between the two different types of evolutionary changes in host specificity [3]. Host switch may be defined as a sudden, often accidental, jump and colonization of a new host species by a few parasite individuals that are able to establish a new and viable population there. After the switch, the new population is more-or-less isolated from the parasite population in the donor host species. It does not affect the further fate of the conspecific parasites in the donor host, and it may finally lead to parasite speciation. Past host switches within a group of parasites are often inferred from a comparison of the parasite phylogeny with that of the hosts. Congruence between the phylogenies is often attributed to a history of association by descent with co-speciation, and incongruence to host-switching or extinction in 'duplicated' parasite lineages, which diverged without a corresponding branching of the host tree. Examples: HIV; SARS, and possibly Guinea worm (if dogs represent a new key host rather than a previously undetected reservoir).
host shift	host shift may be defined as a gradual change of the relative role of a particular host species as key versus subsidiary host, in the case of a multi-host–parasite species. The former primary key host slowly becomes a secondary host or even becomes totally abandoned, while the former secondary host becomes a new key host species. This process is generally slower than host switches. Potential examples: Guinea worm? If dogs were always reservoir hosts but simply previously not reported.
hybridization	from a taxonomic perspective, hybrid refers to offspring resulting from the interbreeding between two animal species or plant species—usually between species in the same genera. An intra-specific hybrid may refer to crosses between subspecies or different populations of the same species.
intermediate host	a host that harbours, and transmits, the pathogen at a larval or asexual stage [2]. Usually, some form of developmental stage is completed in which the pathogen may multiply asexually but not sexually. Intermediate hosts may be vertebrates or invertebrates.
introgressive hybridization	introgression, also known as introgressive hybridization, in genetics is the movement of a gene (gene flow) from one species into the gene pool of another by the repeated backcrossing of an interspecific hybrid with one of its parent species. Introgression is an important source of genetic variation in natural populations and may contribute to adaptation and even adaptive radiation. Introgression differs from simple hybridization. Introgression results in a complex mixture of parental genes, while simple hybridization results in a more uniform mixture, which in the first generation will be an even mix of two parental species.
key host	host species (where host here also includes intermediate hosts and/or vector species) that individually contribute significantly to long-term parasite persistence and drive infection risk in sympatric host species relative to other host species. Key hosts can arise through different mechanisms (i.e. super-abundant, super-infected or super-shedding), which may be due to innate differences among the species (i.e. genetic compatibility), co-infection by other parasite species facilitating infection and transmission by the focal parasite, or may even arise through behavioural modification by the parasite to facilitate super-infectivity. These different types of key host can have important implications for the optimal targeting of control. Hence, not only identifying key host species, but identifying which kind of key host species they are, is imperative for optimal targeting of control strategies.
mode, route versus pathway of transmission	modes of pathogen transmission between infected individuals and susceptible hosts may be ‘direct’, via vertical (including cytoplasmic, transplacental, during vaginal birth or breast feeding), direct physical contact (body surface to body surface), sexual or inoculation/blood-borne transmission, or ‘indirect’, via aerosol/airborne, vector/intermediate-host-borne, fomites/vehicle-borne, water and food-borne pathways. The broader term ‘transmission pathway’ is also often used, particularly in the context of ‘risk analyses’ [4]. Transmission pathway encompasses both the mode by which the pathogen leaves one host and enters the next, for example, faecal–oral, and the specific route it takes.
mode switch	as for host switch and host shift above, although often difficult to disentangle without long-term host–parasite data, it may be important to distinguish between the two different types of evolutionary changes. Mode switch may be defined as the adaptation towards a novel transmission mode by a few individuals that are capable to establish a new and viable interhost transmission pathway. Potential example: *Treponema pallidum* from direct contact in endemic syphilis to sexual-borne in venereal syphilis.
mode shift	where minor transmission modes could become major pathways given new circumstances and opportunities. Potential examples: Zika virus from vector-borne to sexual (semen)-borne.
*R*_0_, the basic reproduction number	a pathogen's fitness can be measured by its basic reproduction number, or *R*_0_. For microparasites, *R*_0_ is defined as the number of new infections arising from a single primary infection in a wholly susceptible host population or community; for macroparasites, *R*_0_ can be defined as the average number of offspring (or female offspring in the case of dioecious parasites) produced from an adult parasite that themselves reach reproductive maturity in the absence of density-dependent constraints on population growth [5]. This definition provides a threshold for parasite invasion into a naive host population; if *R*_0_ > 1 then the parasite can invade, if not then the parasite cannot. In deterministic models, this also equates to the condition for pathogen persistence within that host population. In a multi-host species context, the overall *R*_0_ of the pathogen within the community (*R*_0,TOT_) depends on the combined contributions, *R*_0,*i*_, of each host species *i* [6,7].
reservoir host	one or more epidemiologically connected populations or environments in which the pathogen can be permanently maintained and from which infection is transmitted to the defined target population [8].
vector	at its simplest, often an invertebrate animal that actively transmits an infectious agent between infected and susceptible vertebrates, without undergoing a stage of development or multiplication. In addition, vectors may be able to pass the agent on to their own offspring transovarially. *Transovarial* transmission enables an infectious agent to be maintained in a vector population through many generations without that population having to be reinfected, and, as such, the vector population remains a continuous source of risk.

**Table 2.** Key parameters determining transmission dynamics of multi-host and multi-transmission mode systems (as shown in figure 3), empirical approaches for their estimation, and interventions and other anthropogenic pressures which may influence them.

parameter in figure 3	definition	factors influencing/dictating	empirical approaches for estimation	possible interventions	other anthropogenic selective pressures
*S_i_*(*t*), *I_i_*(*t*)	number of susceptible and infectious individuals of species *i* at time *t*	relative abundance and density of each host species, host population dynamics and movements (births and migration, not shown in model schematics). Dynamics determined by other parameters in the table	epidemiological surveys/surveillance (see table 3, key question 1)	vaccination, population control/culling of non-human hosts	urbanization, migration, land use change, climate change, livestock intensification
*E* (*t*)	infective stages in the environment, vector or intermediate host	dynamics determined by other parameters in the table	environmental sampling, vector/intermediate host surveys	environmental modification/disinfection, sanitation measures, vector/intermediate host control	urbanization, land use change, climate change
*β_ij_* (figure 3*a*)	*per capita* transmission rate from infectious host of species *j* to susceptible host of species *i*	contact rate within species (*j* = *i*) and between species (*j* ≠ *i*); probability of transmission given contact	contact rate surveys, host range mapping, comparative studies of pathogen shedding rates across host species and transmission modes, pathogen population genetics, model fitting to epidemiological data, interviews/outbreak investigations, risk factor studies, experimental studies (see table 3, key questions 2–6)	social distancing, quarantine/isolation, health education, biosecurity measures sanitation, meat inspection, food hygiene, bed nets, vector/intermediate host control, environmental modification	urbanization, migration, land use change, livestock intensification
*β_k_* (figure 3*b*)	*per capita* rate at which susceptible hosts become infected via transmission mode *k*	as above. Also for indirect modes of transmission: host exposure rate to environmental source/vector/intermediate hosts			
1/γ*_i_*	average duration of infectiousness for host of species *i*	host recovery and/or mortality rates	shedding studies, experimental infections, clinical observations (see table 3, key question 5)	medical treatment, mass drug administration	co-infecting pathogens (e.g. via impact on pathogenicity and/or immune response)
*α*	rate of environmental contamination or transmission to vector/intermediate hosts	parasite burden, shedding rates, concentration of pathogen in excretions, e.g. faeces/urine, vector biting rates	shedding studies, model fitting to epidemiological data (see table 3, key questions 4–6)	anti-fecundity vaccination (e.g *Schistosoma japonicum*), environmental modification, sanitation infrastructure (e.g. latrines), physical barriers (e.g. bed nets), health education	co-infecting pathogens (e.g. via impact on pathogenicity and/or immune response)
*θ*	decay rate of infective stages in the environment/vector/intermediate hosts	biological properties of pathogen, environmental factors, population biology of vector/intermediate hosts	environmental persistence studies, vector/intermediate hosts studies	environmental modification/disinfection, vector/intermediate host control	land use change, climate change

The goals of many disease control programmes, including those targeting pathogens with multiple hosts and/or transmission modes, are increasingly shifting towards elimination or even, in certain cases, eradication [14,15] (table 1). Examining how pathogens respond to such strong anthropogenic changes as those imposed by these interventions offers unique opportunities for ‘quasi-experimental studies’ in adaptive management frameworks and can play a crucial role in enriching our mechanistic understanding of transmission dynamics under contrasting selective pressures [16]. Disentangling the transmission dynamics of the infecting agent/s is particularly important, not only to identify key hosts and modes against which interventions could or should be targeted, but also to anticipate potential unintended consequences (positive and negative) that may occur in response to the selective pressures that elimination efforts exert on these systems.

Here, we explore, focusing on examples from both human and animal pathogen systems, how the complexities of multi-host multi-mode infectious agent transmission dynamics may challenge our abilities to understand and predict disease dynamics, why and how we should aim to disentangle and quantify their relative importance under contrasting conditions, and ultimately, how this can be used to help achieve efficient and effective disease control.

## 2. Multiple hosts, pathogens and modes of transmission

### (a) Multiple host species and stages

Most diseases globally involve multiple host species [17,18], with an estimated 60–75% of newly emerging diseases in humans being multi-host zoonoses, i.e. infectious diseases that are naturally transmitted between vertebrate animals and humans [18,19]. Many multi-host infectious agents have the additional feature of a complex, indirect life cycle, where different life stages of a pathogen are found in often highly unrelated phylogenetically, definitive and intermediate (and/or secondary or vector) host species (table 1). For example, many trematodes have both obligatory mammalian and avian definitive host stages, as well as a molluscan intermediate host stage. The epidemiology of such multi-host infectious agents thereby involves multi-faceted communities of definitive host species and individuals, together with vector or intermediate species and individuals, all of which may represent potential targets, as well as specific challenges, in the context of disease control, particularly where elimination is proposed [8,12]. However, the majority of epidemiological theory to date has focused on a single-pathogen single-host framework [20]. Even for zoonoses, if the disease is considered to be of no economic importance or is asymptomatic in animals, humans historically have generally been the only species considered when designing control programmes. In multi-host systems, a failure to understand or at least consider the potential importance of other animal hosts when planning interventions may mean control efforts are ineffective or at best inefficient.

In diseases with only one host species, the force of infection, defined as the instantaneous hazard or risk experienced by a susceptible individual, is likely to be predominantly dictated by a combination of the number or proportion of infectious individuals in the population (depending on whether transmission is density or frequency-dependent), contact rate between individuals, probability of transmission given contact and the duration of infectiousness. This becomes more complicated when multiple hosts are involved in transmission, as each host species or stage is unlikely to contribute equally to the force of infection due to heterogeneities and trade-offs in these parameters across species and stages [6,8,21,22]*.* Even infectious agents with a very broad host range are often transmitted predominantly by just a subset of potential hosts, or key host species (table 1), and this may vary in different contexts or ecosystems. Rabies virus, for instance, is a pathogen with the potential to infect all mammals, but its long-term persistence in an ecosystem typically depends on a maintenance key host, usually a carnivore or bat species [23]. For example, in the Serengeti ecosystem, rabies transmission maintenance appears to be dependent on domestic dogs [24].

Behavioural patterns may play a role in determining the importance of potential hosts within a system, and hence, key hosts may not necessarily be highly abundant but have a behavioural repertoire that places them in high contact with other suitable host species, for example, the roosting behaviour and habitat selection of bats and their link to Nipah virus epidemiology [25]. Certain pathogen species also have behavioural patterns to maximize their opportunities for transmission to key host species. The larval propagule stage of *S. japonicum* in China, for example, shows different behavioural (and genetic) profiles in relation to the key maintenance host species present: in hilly regions where nocturnal rodents are the species which predominantly maintain transmission, cercariae are shed from *Oncomelanaia* snails in the late afternoons and evening, whereas in lowland habitats where bovines drive transmission, early morning shedding occurs, coinciding with the timing of peak bovine water contact [26,27]. Even more intriguing are cases where certain complex life cycle pathogens manipulate their hosts' behaviour to facilitate transmission from one host species and stage to another, and there are numerous cases within parasitized invertebrates [28]. Examples of specific manipulation of vertebrate host behaviour are rarer, although increased aggression is proposed to enhance transmission, via blood and/or saliva through biting, of viruses such as rabies, Hantaan and Seoul [29]. *Toxoplasma gondii* appears to enhance the likelihood of rodent intermediate hosts being preyed upon by their feline definitive hosts through subtle manipulation of a whole suite of predator-risk behaviours [30–40]. Moreover, *T. gondii* appears to subtly alter the rats' cognitive perception of predation risk, turning their innate aversion to predator odour into a ‘suicidal’ ‘fatal feline attraction’ and this appears specific towards their feline definitive host [40–42]. There do, however, appear to be differences between domestic and wild species of felines, potentially in relation to their capacities as efficient definitive hosts [43].

### (b) Multiple modes, routes and pathways of transmission

The terms transmission ‘mode’, ‘route’ and ‘pathways' are often used interchangeably and the terminology can be confusing (discussed in [20]) as well as varying between public health and evolutionary biology literature. In terms of disentangling pathogen transmission dynamics and identifying where and when to target control programme activities, the level of resolution is likely to be important.

Modes of pathogen transmission between infected individuals and susceptible hosts may be ‘direct’, via vertical (including cytoplasmic, transplacental, during vaginal birth or breast feeding), direct physical contact (body surface to body surface), sexual or inoculation/blood-borne transmission, or ‘indirect’, via aerosol/airborne, vector/intermediate-host-borne, fomites/vehicle-borne, water and food-borne pathways (figures 1 and 2). Within the evolutionary literature on disease, a major distinction between transmission modes, particularly in terms of the evolution of virulence [20,44], has been between ‘vertical’ (as above) and ‘horizontal’, which encompasses both direct and indirect modes. The broader term ‘transmission pathway’ is also often used, particularly in the context of ‘risk analyses’ in relation to, among other issues, food-borne diseases/food safety [4]. The transmission pathway in this context is the sequence of steps needed for the undesirable outcome (i.e. exposure/infection of the host) to occur. Transmission pathway thereby encompasses both the mode by which the pathogen leaves one host and enters the next, for example, faecal–oral, and the specific route it takes, for example, via a fomite or via water contamination. *Toxoplasma gondii*, for instance, may be transmitted to a susceptible host through the indirect food-borne mode, but in terms of managing risk or implementing control strategies, it is important to differentiate between the different possible food-borne routes through which the host may have been infected. The new host will have eaten infected meat, but the meat could have been either from an infected animal (i.e. with *T. gondii* bradyzoites) or the animal was not infected, but there was contamination of the food product at some stage (e.g. with *T. gondii* oocysts). Thus, in this example, the transmission pathway encompasses different routes but the same mode of transmission. Conceptualizing exposure in this way is convenient as it allows an overall evaluation of risk of exposure by combining the probabilities (*P*) of the series of events occurring, for example: *P* (animal is infected) × *P* (infected animal is not detected and removed from the food chain) × *P* (viable pathogen is present in the meat of infected animal) × *P* (pathogen not inactivated by processing) × *P* (food with viable pathogen consumed by a susceptible person). By decomposing transmission into multiple steps, it may be possible to intervene with control measures and evaluate effects at different levels.

Disentangling transmission dynamics becomes even more complex, however, as many infectious agents have the potential to be transmitted to susceptible individuals via more than one mode of transmission and pathogens may use all possible transmission modes simultaneously or even switch according to conditions [20]. For example, Rift Valley fever virus (RVFV) is usually transmitted among livestock, specifically cattle, sheep and goats, via mosquitoes bites, but can also be transmitted vertically between animals, even in the absence of detectable maternal viraemia [45]. Transmission of RVFV from domestic animals to humans occurs mainly through direct contact with blood, excreta, meat, milk or other secretions of infected animals, but in a few cases, zoonotic transmission can also occur through mosquito vectors [46,47]. It is unclear which, if any, animal species maintain RVFV during the wet seasons and interepidemic periods, but it is believed that RVFV can be maintained during these periods solely within the mosquito population via alternative transmission pathways, including via transovarial vertical transmission within certain mosquito species [48].

Another classic example is *T. gondii*. While having only one definitive host, a member of the Felidae, which shed oocysts within the stool, all warm blooded organisms can become infected by this protozoan, either via the consumption of vegetation or water contaminated with the highly resistant oocysts or by consuming raw or undercooked meat containing bradyzoite cyst stages. Moreover, in spite of causing substantial abortion or mortality in certain secondary host species such as sheep and humans, some species, in particular mice and rats, appear to maintain infection through congenital or neonatal transmission [49–51]. Several cases of successful sexual transmission, many with consequent vertical transmission to their progeny, have also been documented in experimental studies involving, but not exclusive to, rats [52], dogs [53], sheep [54,55] and goats [56,57]. Sexual transmission through *T. gondii* tachyzoites in semen has also been proposed as a potential transmission mode for human toxoplasmosis [58,59], but it remains unknown how prevalent or successful these different modes are under natural conditions.

Such a multiplicity of modes, routes and pathways through which a pathogen can spread presents additional challenges during disease outbreaks in terms of identifying the source or sources of infection. Foot-and-mouth disease (FMD) virus, for example, which causes an acute vesicular disease of domesticated and wild ruminants and pigs, can be spread through the movements of infected animals or their bodily fluid, faeces, urine, contaminated persons, objects and aerosols [60]. While some host species, such as cattle and sheep, are believed to be primarily infected through respiratory modes such as aerosol, other potential host species, such as pigs, are believed to be more likely to be infected through wounds or ingestion [61]*.* Furthermore, some species can serve as carriers of FMD, remaining infectious for up to 5 years [62]. Transmission can be further amplified through anthropogenic means such as vehicles and humans serving as mechanical vectors, as well as via environmental waterways and animal products. The multiple potential transmission pathways of this persistent disease have repeatedly served to complicate FMD outbreak control and prevention strategies [63].

Considering all potential modes, routes and overall pathways of transmission is, therefore, imperative when it comes to planning or implementing disease control interventions. However, we often know so little about their relative importance or the forces of selection acting on them at different times.

### (c) Dynamic hosts, pathogens and pathways

An additional challenge for disease control or elimination is the capacity of pathogens to evolve in the face of changing pressures, which may mean, for instance, an alteration in or expansion of the key hosts and host range within a system, or even an alteration or expansion of the transmission modes and pathways available.

Host switches, whereby a pathogen successfully jumps from one host species to another (table 1), are thought to have been a major process in the evolution of many infectious agents and can be an unpredictable consequence of the changing evolutionary pressures, including those exerted by disease control interventions. Biological and epidemiological features of the disease, modes of transmission and host susceptibility can all influence an infectious agent's ability to switch host species [64,65]. Pathogens, particularly those with high mutation rates, antigenic diversity and short generation times, may rapidly adapt to new host species [66–68] and evidence suggests that RNA viruses are the most likely group of infectious agents to switch hosts and establish in humans [1]. This is illustrated by influenza A viruses, for which avian and swine hosts are the main reservoirs. Sporadic human infections with zoonotic influenza viruses are well documented, particularly for avian influenza subtypes A/H5N1 and, more recently A/H7N9. Human-to-human transmission is typically limited following these spillover events, but genetic re-assortment between influenza strains within co-infected humans, birds or pigs, and acquisition of human-specific respiratory epithelium receptors, can lead to novel, human-adapted strains with pandemic potential [69]. Similarly, canine distemper virus (CDV) is also an RNA virus with global distribution and an expanding range of host species, including domestic and wild canids, marine mammals, felids, procyonids and ursids, and non-human primates. The propensity of CDV for host-switching has raised concerns about both potential risks for humans and extinction threats to endangered wildlife [70].

The strength of the selective pressures imposed upon the pathogen will also impact its likelihood to switch and adapt to new host species. There are numerous examples where agricultural intensification and environmental change have been proposed as key anthropogenic drivers for zoonotic disease emergence (reviewed in [71]), but pressures exerted by control interventions themselves could also lead to host or transmission mode shifts. An important potential example is Dracunculiasis, caused by the Guinea worm *Dracunculus medinensis*, that has been targeted for eradication since the early 1990s [72]. Dracunculiasis was rediscovered in Chad in 2010 after an apparent absence of human cases for 10 years, and it appears that dogs may now serve as keys hosts for sustaining transmission in this setting, with potentially an additional aberrant life cycle pathway involving a paratenic host involved in ongoing transmission to both humans and dogs [73,74]. This particular example may also, therefore, highlight the potential for interdependencies between switches and/or shifts in host species and transmission pathways.

Host-switching also enhances opportunities for novel interactions between multiple infectious agents in co-infected individuals. Co-infecting pathogens can have profound effects on pathogen ecology and evolution, both through direct interpathogen interactions and/or via the host's immune response [75–77]. A particular challenge regarding elimination of multi-host pathogens is the phenomenon of hybridizations and introgressions (table 1), which can contribute to adaptation and even the expansion of key host range [78,79]. Evidence for hybridizations and introgressions between a broad range of pathogen species is gathering, partly in line with improvements in molecular diagnostics and genome sequencing of these organisms [12,13]. One example is schistosomiasis in West Africa, where it had previously been thought that the human and animal schistosomes were separate, and control and surveillance efforts have subsequently focused entirely on the human population alone. However, molecular techniques have revealed that within certain regions, a large proportion of both the human definitive and the snail intermediate host populations are infected with introgressions between the human schistosome species *Schistosoma haematobium* with the ruminant species *Schistosoma bovis* and/or *Schistosoma curassoni* [80,81]. This raises the important question of whether, at least in certain settings in Africa, the role of non-human mammalian hosts in the transmission dynamics of human schistosomiasis has been severely underestimated.

Mode switches, whereby a pathogen successfully switches to a new mode of transmission (or mode shift, whereby a pathogen successfully alters the predomination of one mode to another; table 1), in contrast with that of host switches and shifts, have rarely been documented in the evolutionary and disease literature. Of the few, in addition to the *T. gondii* in rodents example cited above [49], there is evidence from the 1991 cholera epidemic in South America that *Vibrio cholera* can shift towards predominantly foodborne transmission modes under conditions of and in countries with high sanitation, while its more virulent waterborne mode predominates under conditions of poor sanitation [82,83]. It has also been proposed that the endemic syphilis may have switched mode from the direct skin contact mode, usually transmitted during childhood, of the endemic syphiles (*Treponema pallidum* subsp. *pertenue*, the causative agent of yaws, and *T. pallidum* subsp. *endemicum*, the causative agent of bejel) in tropical developing countries to the sexually transmitted mode of venereal syphilis (*T. pallidum* subsp. *pallidum)* in temperate developed countries. The original ‘unified’ theory proposed that all three treponemal diseases were caused by the same aetiological agent and that the mode of transmission and clinical characteristics of infection were dictated by the environment and opportunities [84]. There are recent sequencing data both in support (and contradiction) of this [85]. However, recent studies have also identified, for example, cases of venereal syphilis in temperate counties caused by the yaws subspecies [85]. Thus, these treponemes may be potentially indicative of dynamic mode shifts rather than true mode switches under contrasting environments and pressures. Even more intriguing perhaps is recent evidence of *Treponema* subspecies hybridization, which could be hypothesized to further enhance the potential for multiple-mode transmission dynamics [13,86]. There are current fears and gathering evidence that Zika virus may also increase and/or continue to be transmitted, despite increased vector control, through a mode switch (or shift) towards sexual transmission [87,88]. Similarly, in the recent Ebola epidemic, there were fears that the Ebola virus might evolve aerosol transmission, given greater opportunities for this mode of transmission in crowded human situations, especially as aerosol transmission of filoviruses has been demonstrated in laboratory experiments [89].

## 3. Disentangling and quantifying transmission

### (a) Conceptualizing and modelling multi-host transmission

Although much epidemiological theory has focused on single-host systems, a number of conceptual frameworks have been put forward to aid our understanding of multi-host–pathogen systems. As with single-host systems, the basic reproduction number, *R*_0_, is often central to these frameworks [6–8], with *R*_0_ being defined as the expected number of secondary infections generated by a typical infectious individual in a totally susceptible population [90]. In particular, for a multi-host–parasite to persist in a system, the overall basic reproduction number across the host community (denoted *R*_0,tot_) must be greater than 1, giving a useful threshold for parasite elimination (i.e. *R*_0,tot_ < 1). Within that system, *R*_0,tot_ will depend on the basic reproduction number within each host species, *i* (*R*_0,*i*_), as well as the level of heterogeneous ‘structuring’ of transmission (that is transmission between host species relative to that within host species, relating to the issue of ‘who acquires infection from whom’ (WAIFW), which we return to below) [6]. Only those host species for which *R*_0,*i*_ is greater than 1 will be capable of independently sustaining transmission in the absence of other host species; these hosts can be referred to as ‘maintenance hosts', using terminology proposed by Haydon *et al*. [8]. If there are several maintenance host species (*R*_0,*i*_ > 1 for more than one host), this can be referred to as a system with ‘facultative multi-host parasitism’. If there are no maintenance hosts (*R*_0,*i*_ < 1 for all hosts) in a system, but a community of hosts can together maintain transmission (*R*_0,tot_ > 1), this can be termed ‘obligate multi-host parasitism’, under the framework proposed by Fenton *et al*. [6]. Another type of key host, termed an ‘essential host’, can be defined as one for which transmission cannot be sustained (*R*_0.tot_ < 1) in the absence of its contribution to transmission. (Note that the terms maintenance host and essential host are not mutually exclusive but neither are they synonymous.)

Since *R*_0,*i*_ and *R*_0,tot_ cannot be measured directly, they must typically be derived through mathematical models. The structure and assumptions of a multi-host model, and thus the mathematical expressions for *R*_0,*i*_ and *R*_0,tot_ and types of data needed for their estimation, will depend on the specific multi-host–pathogen system under investigation (a generic model of a system with two host species is given in figure 3*a*). In general, however, for a model with *n* host groups, *R*_0,tot_ can be derived from the largest eigenvalue of the *n* *×* *n* next-generation matrix of the model, the elements of which represent the number of new infections in host group *i* generated by a single infected host in group *j* [7,90]. (Thus, the diagonal elements of this matrix, *i* = *j*, represent *R*_0,*i*_.) The elements of the next-generation matrix will depend on: (i) rates of transmission within and between host species, described by the WAIFW matrix; (ii) duration of infectiousness for each host group (and, for indirectly transmitted pathogens, the persistence of infective stages in the environment, vector or intermediate host); and (iii) the relative abundance or density of each host species. (See [7,90] for full details on how the next-generation matrix and *R*_0_ are derived from models with heterogeneous transmission.)

### (b) Empirical approaches for quantifying transmission by host species

While models can help us identify the types of factors that are important for determining multi-host transmission dynamics, empirical data are essential in order to parametrize models and gain quantitative insights into the relative importance of different host species and thus, the potential impact of different interventions (tables 2 and 3). Parameters for duration of infectiousness and host densities (components (ii) and (iii) mentioned above) can often be measured directly. Host population sizes are typically observable for human and livestock populations and, although more challenging, can usually be estimated for wildlife populations using, for example, mark/recapture studies. The duration of infectiousness in each host individual and/or group (which should account for both recovery and mortality rates) can usually be estimated from clinical, veterinary and/or epidemiological data, and where diseases have an environmental source of transmission, such as waterborne infections [83,118,119], persistence of the pathogen in the environment can also often be directly measured [103]. This persistence in the environment can be considered as an extension of the infectious period, a reservoir of the infectious agent or a combination of the two [120], and models of diseases with environmental source of transmission often explicitly include an environmental compartment contributed to by infectious individuals [121] (figure 3*b*).

**Table 3.** Empirical approaches to disentangling multi-host and/or multi-mode transmission.

key question	empirical approaches	examples
1. which hosts are potentially involved in transmission (key hosts)?/which species in the ecosystem are infected?	epidemiological studies, such as seroprevalence, parasitological and/or molecular typing studies from humans and animals can be used to identify potential hosts.	[70,81,91–93]
	comparison of human and veterinary surveillance data can provide early indication that an outbreak of disease in humans may have a zoonotic origin.	[94–96]
2. is there potential for effective contact between host species and, if so, how do contact rates compare between versus within species?	GPS tracking can be used to asses contact between wildlife species and between wildlife and domestic livestock.	[94]
	ecological studies of wildlife hosts can identify potential interspecies transmission pathways to humans.	[25]
3. is there evidence of cross-species transmission and host shifts?	population genomic and genetic studies can type infecting pathogen species and demonstrate gene flow across known host species. Sequence data can be combined within biostatistical and/or mathematical frameworks (e.g. space state modelling) to reconstruct cross-species transmission events. The latter can be particularly useful to also discriminate between recent cross-species transmissions, many of which may result in dead-end infections, and host shifts that reflect successful onwards transmission in the new host species.	[9,81,97–99]
4. what are the potential modes of transmission/transmission pathways?	studies of the presence of pathogen in different body fluids/excreta can identify or confirm zoonotic sources of infections and indicate unconventional or previously unknown transmission pathways aiding the understanding of transmission pathways and providing focus for epidemiological studies.	[100–102]
	experimental infections can demonstrate potential for alternative pathways that may not have been considered, and may identify which modes of transmission are most important.	[49,103,104]
5. which potential host is most infectious?	studies of pathogen shedding by different species, including amount of pathogen spread and duration of shedding can be used to assess the potential relative contribution of different host species to transmission.	[105–109]
6. who is acquiring infection from whom and how?	interviews, contact tracing and risk factor studies can for some diseases indicate how the majority of transmission events are occurring, thereby identifying the most important transmission pathways and enabling targeting of interventions.	[110–112]
	mixing studies, for example, of contact rates between age groups in human populations, can predict which age group would contribute most to spread of infection in a disease outbreak, which can be extremely useful for planning and preparedness, e.g. vaccine stockpiling.	[113]
	molecular techniques such as whole genome sequencing can for some diseases be used to trace transmission events.	[114]
7. which transmission pathway or group is driving transmission, and therefore where should interventions be targeted?	mathematical models of disease dynamics, informed by many of the above forms of study, can be used to identify key and maintenance hosts, and also to predict the impact of interventions.	[6,10,115–117]

The main challenge for quantifying multi-host transmission dynamics typically lies in parametrization of the WAIFW matrix, as the transmission rates, *β_ij_*, within and between species which make up the elements of the matrix again cannot normally be measured directly (see [122]). However, the relative magnitudes of values in a WAIFW matrix will depend largely on the relative infectiousness of each host species and contact rates within and between host groups, on which empirical evidence can, in many cases, be obtained. For example, the relative infectiousness of each species can sometimes be quantified by comparing pathogen shedding rates across host species, as has been achieved through examinations of the relative presence of bovine tuberculosis *Mycobacterium bovis* in the faeces, urine and tracheal aspirates of free-living wildlife in the UK [105], through comparative measurements of the eggs of *S. japonicum* shed per day in the stools of domestic and wild animals in China [106,123], and likewise comparative measurements of *T. gondii* oocysts shed per day in the stool of domestic and wild cats [107,108]. Heterogeneities in levels of infectiousness within, as well as between, host species can also be important to consider, given that parasite aggregation among hosts and the potential for ‘super-spreaders’ are common phenomena that can have important implications for disease dynamics and control.

In terms of measuring contact rates, at least within human populations, this can be done through questionnaires and contact diaries, for example, to identify age-assortative mixing patterns [113,124–126]. However, a contact that has the potential to effectively transmit infection can be hard to define, and will vary between diseases. Interhost species mixing patterns, particularly between animal populations, can be even more challenging to measure, although if largely dependent on spatial structuring can be inferred from degree of overlap in host ranges or habitats, as was done in a modelling study to identify key animal reservoirs of African trypanosomiasis [115]. Technological advances such as video-capture, radio-tracking and GPS tracking have also provided useful insights into wildlife population contact rates, both within species, for example, deer [127], and between species, such as in study on risk of Hendra virus transmission between flying-foxes and horses in Australia [94].

Evidence to inform relative rates of transmission between versus within species can also be obtained through molecular epidemiological approaches. For clusters of avian influenza infections in humans, the relatedness of virus genomes between cases can help ascertain whether any cases with no history of exposure to sick poultry may represent human–human transmission events [128]. Meanwhile, population genetics studies of schistosomiasis have been used to estimate levels of parasite genetic differentiation across host species in China and the Philippines, to give at least qualitative insights into the degree of transmission structuring between hosts [9,129]. Novel phylogenetic tools are increasingly being used to assessing rates and directionality of interspecies transmission, for example, of bovine tuberculosis [97] and rabies [98], while advances in phylodynamic approaches, in which transmission models are directly fitted to observed pathogen phylogenies, also show much promise [65,130].

The types of empirical data to inform WAIFW matrices mentioned above, such as on the contact patterns and infectiousness of different host species, will allow transmission rates to be scaled between versus within species. However, one cannot usually calculate the actual magnitude of β parameters from such data alone; typically, this will be done indirectly through fitting the model to epidemiological data collected across host species. For endemic diseases, if it can be reasonably assumed that dynamics are at a steady-state equilibrium, cross-sectional prevalence data across host species will be sufficient. For example, in the case of the multi-host zoonotic parasite *S. japonicum*, relatively straightforward epidemiological and parasitological data allowed the different potential host species contributions to *R*_0,tot_ to be quantified, and important conclusions about transmission and the likely effects of control measures to be made [10].

For outbreaks or emerging diseases, estimation of transmission rates and *R*_0_ will probably require the model to be fitted to longitudinal data. The difficulty here is that surveillance and reporting of animal diseases is often poor, especially in wildlife but also in livestock diseases in many countries. For many diseases with animal reservoirs of infection, occasional spillover into the human population is often the only indication of ongoing and poorly understood epizootic or enzootic transmission, as we have seen with outbreaks of Ebola [131] and Nipah virus [99].

### (c) Quantifying transmission by transmission modes and pathways

Conceptually, at least, extending a model to consider multiple transmission pathways (encompassing the alternative potential modes and routes of infection) within and between host species is relatively straightforward. This can be done by partitioning each element of the WAIFW matrix, *β_ij_*, by transmission mode *k*, such that the rate of infection from species *j* to species *i* can be defined as:

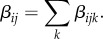
The next-generation matrix for the model, and thus *R*_0,tot_, can then likewise be partitioned by each transmission mode *k*, in addition to each host species *i*. Thus, the concepts for multi-host–pathogen systems described above can similarly be applied to multi-mode systems, with transmission mode-specific *R*_0_ values (*R*_0,*k*_) providing a basis from which to identify ‘maintenance’ and ‘essential’ transmission modes, and differentiate between obligate versus facultative multi-mode systems. (We should also note that, depending on the system under investigation, *k* could also represent different pathways if, for a given transmission mode, there are multiple routes the pathogen might take which should be considered separately.)

The real challenge, once again, lies in obtaining sufficient empirical evidence to parametrize the models and quantify the relative importance of different transmission modes. Nevertheless, there are approaches through which such evidence can be collected (tables 2 and 3). For example, the rate and duration of pathogen excretion and environmental persistence via different modes can, in principle, be measured. Examples include the recently reported prolonged shedding of Ebola virus in semen [100], and studies on duration of environmental persistence and infectivity of avian influenza virus via aerosol and faecal–oral modes [103]. For humans, behavioural surveys and classical epidemiological risk factor studies can be useful in determining the relative frequency of and risks associated with different types of exposure. In the case of rabies, medical records and verbal post-mortems will often provide information on history of an animal bite and, therefore, which species most likely transmitted infection [110]. For human cases of highly pathogenic avian influenza, case investigations and interviews have been useful in identifying which types of exposure to sick poultry may carry the greatest risk for zoonotic transmission [132]. In the case of sexually transmitted infections, such as HIV, specific types of contact can be defined and measured, to enable estimates of the probability of transmission per act and by type of act [133]. In the few diseases where different forms of exposure are associated with different disease courses, surveillance and clinical data during or after an outbreak can also be used to identify most likely sources of transmission and guide further epidemiological investigations. Examples include anthrax, which has distinct clinical symptoms for different forms of exposure (inhalation, ingestion or cutaneous), and *Yersinia pestis* where flea bites are more likely to cause the bubonic form, whereas the pulmonic form can be transmitted directly from human to human [134].

As with multi-host transmission dynamics, genetic and/or genomic data can also provide important insights into the relative importance of different modes and pathways. For example, some modes of transmission may tend to involve a larger pathogen inoculum dose than others (e.g. ingestion of a heavily contaminated food source compared with aerosol infection), for which one may expect to observe higher intra-host microbial diversity [135]. For livestock diseases, the reconstruction of interfarm outbreak spread based on phylogenetic and epidemiological data, along with data on factors such as animal and human movements, road networks, wind direction and distance between farms, can give insights into the potential role of different interfarm transmission pathways (e.g. wind- versus human-mediated transmission) [136,137].

One advantage in the case of animal diseases is the possibility to use experimental infections to inform estimates of probability of transmission for different forms of exposure, and the relative importance of different transmission routes. For avian influenza, studies have involved exposing susceptible birds to experimentally inoculated birds in such a way that either only aerosol or only faecal–oral transmission could occur [103,138,139]. Similarly, experimental studies on FMD virus have been used to estimate the relative importance of direct versus indirect transmission on farms, by exposing groups of susceptible calves either directly to infected individuals or by housing them in buildings that had previously held inoculated individuals [104]. A semi-naturalist captive study examining mode of transmission of *T. gondii* in wild brown rats, *Rattus norvegicus*, in the UK aimed to determine if the congenital transmission route alone could be successful and sufficient at maintaining transmission [49]. The study found that, in the absence of oocyst (faecal) contamination from the feline definitive host or bradyzoite exposure through contaminated meat, the seroprevalence remained stable over several generations of rats, suggesting that congenital transmission might be a ‘maintenance’ transmission mode for *T. gondii*. However, other modes of transmission, such as cannibalism, sexual transmission or even importation of oocysts into the enclosure by paratenic hosts (e.g. earthworms), could not be fully ruled out, illustrating the difficulty of controlling all possible transmission modes even in experimental studies.

## 4. Implications for disentangling transmission in the ‘elimination era’

We live in a time where disease ‘elimination as a public health problem’ and even ‘eradication’ have been proposed as Millennium Development Goals and more recently, the Sustainable Development Goals [15,140]. These goals are difficult to achieve for any infectious disease, as reflected by the fact that only one human and one animal pathogen (smallpox and rinderpest, respectively) have been globally eradicated to date [141]. The distinct biological features of different infectious agents and the technical factors for dealing with them make their potential eradication or elimination more or less likely. Three indicators may be considered to be of primary importance: an effective intervention is available to interrupt transmission of the agent; practical diagnostic tools with sufficient sensitivity and specificity are available to detect levels of infection that can lead to transmission; and a single-host species, be it human or animal, is essential for the life cycle of the infectious agent, which has no other vertebrate reservoir and does not amplify in the environment. In addition, the importance of socio-economic and political context (including factors such as health system infrastructure, intersectoral cooperation, financial resources, political will and public acceptance to ensure effective implementation of interventions) in determining the success of elimination programmes must be stressed.

The challenges of elimination are magnified for multi-host and undoubtedly even more so for multi-mode pathogens. Interventions may need to identify and target multiple host species, and/or block or manipulate available transmission pathways [83,118]. For instance, *Brucella melitensis* causes febrile disease in humans and production losses/morbidity in both small (sheep and goat) and large (cattle) ruminants in many parts of the world. Vaccination of sheep and goats alone is, however, the mainstay of current control programmes. Recent mathematical models suggest that the current practice of limiting vaccination to sheep/small ruminants alone would take 16.8 years to achieve elimination on a mixed-species *B. melitensis*-endemic farm, but combining this with cattle vaccination would reduce the time to 3.5 years [142].

The set of tools required for control are also likely to be more diverse for those pathogens for which multiple host species and/or multiple transmission modes exist. Such infectious agents may, for instance, show genetic diversity across different host species, such that a vaccine or drug effective in one host species may not be in another [12]. Drug treatment of animal reservoirs, even with different drugs to those used in humans, may also lead to the development of cross-resistance, rendering human drug treatment less effective [143]. Social and economic challenges may also be specific to, or amplified for, pathogens with multiple hosts and/or transmission pathways. For instance, livestock owners may feel disinclined to report disease in their animals (especially if it may lead to culling), or to treat/or vaccinate their animals, if there is a risk and/or insufficient compensation or perceived benefit from such measures [144].

An additional challenge in multi-host and multi-mode systems in the context of elimination is the capacity of pathogens and transmission dynamics to evolve and change in the face of changing pressures, which may mean an alteration in the key hosts within a system, an expansion of host range and/or an expansion or opportunities for transmission. It remains a matter of urgency to determine with confidence whether new transmission modes (mode switches) may evolve in extant disease threats, or if currently minor transmission modes could become major modes (mode shifts), given new circumstances and opportunities [20].

## 5. Conclusion

Pathogens which have the capacity to be transmitted by multiple hosts and/or via multiple modes may pose the greatest challenge when it comes to disease control and ultimately elimination. Identifying those key hosts and transmission pathways, and thus where interventions would most effectively be targeted, is not straightforward, but important insights can be gained through continued application and development of theoretical and empirical approaches for disentangling transmission dynamics, such as those presented above. Interventions need to be meticulously designed, implemented and monitored to optimize the immediate short-term benefits to the target population(s). Given that such pathogens might be especially able to adaptively switch hosts and transmission modes, particularly in our current era of profound and rapid anthropogenic change, advancing our understanding of evolutionary, as well as ecological, dynamics of multi-host and multi-mode pathogens is also crucial for anticipating and maximizing the ongoing success of elimination programmes.

## Data accessibility

This is a review article and does not include primary data.
